# The Transmembrane Region Is Responsible for Targeting of Adaptor Protein LAX into “Heavy Rafts”

**DOI:** 10.1371/journal.pone.0036330

**Published:** 2012-05-25

**Authors:** Matous Hrdinka, Pavel Otahal, Vaclav Horejsi

**Affiliations:** Institute of Molecular Genetics, Academy of Sciences of the Czech Republic, Prague, Czech Republic; Hungarian Academy of Sciences, Hungary

## Abstract

**Background:**

The importance of membrane compartmentalization into specific membrane microdomains has been shown in many biological processes such as immunoreceptor signaling, membrane trafficking, pathogen infection, and tumor progression. Microdomains like lipid rafts, caveolae and tetraspanin enriched microdomains are relatively resistant to solubilization by some detergents. Large detergent-resistant membrane fragments (DRMs) resulting from such membrane solubilization can be conveniently isolated by density gradient ultracentrifugation or gel filtration. Recently, we described a novel type of raft-like membrane microdomains producing, upon detergent Brij98 solubilization, “heavy DRMs” and containing a number of functionally relevant proteins. Transmembrane adaptor protein LAX is a typical “heavy raft” protein. The present study was designed to identify the molecular determinants targeting LAX-derived constructs to heavy rafts.

**Methodology/Principal Findings:**

We prepared several constructs encoding chimeric proteins containing various informative segments of the LAX sequence and evaluated their effects on targeting to heavy rafts. Replacement of the polybasic membrane-proximal part of LAX by CD3ε-derived membrane-proximal part had no effect on LAX solubilization. Similarly, the membrane-proximal part of LAX, when introduced into non-raft protein CD25 did not change CD25 detergent solubility. These results indicated that membrane-proximal part of LAX is not important for LAX targeting to heavy rafts. On the other hand, the replacement of transmembrane part of CD25 by the transmembrane part of LAX resulted in targeting into heavy rafts. We also show that LAX is not S-acylated, thus palmitoylation is not involved in LAX targeting to heavy rafts. Also, covalent dimerization was excluded as a cause of targeting into heavy rafts.

**Conclusions/Significance:**

We identified the transmembrane domain of LAX as a first motif targeting transmembrane protein constructs to detergent-resistant heavy rafts, a novel type of membrane microdomains containing a number of physiologically important proteins.

## Introduction

According to a presently widely accepted model, the plasma membrane is naturally organized into several types of microdomains differing in their lipid and protein composition and resistance to solubilization by detergents (e.g. Triton X-100, CHAPS, Brij series, dodecylmaltoside). The best defined of these membrane entities are membrane (lipid) rafts [1], caveolae [2], and tetraspanin enriched microdomains (TEMs) [3]. Upon solubilization by e.g. Triton X-100, CHAPS, or Brij series detergents membrane rafts produce “detergent-resistant membrane” (DRM) fragments which, due to their low buoyant density, can be conveniently isolated by density gradient ultracentrifugation [4]. Membrane rafts are, however, readily solubilized by alkylgycosidic type of detergents (octylglucoside, dodecylmaltoside). Membrane rafts and signaling molecules associated with them are thought to play important roles in immunoreceptor signaling [5], [6], [7].

One of the key signaling molecules present in T cell DRMs corresponding to lipid rafts is the transmembrane adaptor protein (TRAP) LAT [8]. Previous results indicated that the palmitoylation-dependent targeting of LAT into membrane rafts is essential for its signaling function [9], [10]. However, a study by Zhu et al. demonstrated that a LAT construct absent from membrane rafts is actually able to support T cell receptor (TCR) signaling and development of T cells *in vivo* [11]. This finding casted doubts on the real physiological role of rafts in TCR signaling. Recently, we demonstrated that these results could be explained by the existence of a novel type of membrane raft-like microdomains, producing upon detergent solubilization “heavy DRMs” [12]. We showed that these microdomains (provisionally called “heavy rafts”) share some properties with classical membrane rafts (resistance vs. sensitivity to particular detergents, cholesterol dependence) but in contrast to them, upon solubilization by Brij98 they produce DRMs of higher density not floating up during density gradient ultracentrifugation. Moreover, they contain a different set of proteins, and are more dependent on protein-protein interactions. According to our results, the chimeric construct used by Zhu et al. [11] composed of cytoplasmic part of LAT and extracellular and transmembrane parts of another TRAP, LAX (constitutively present in heavy rafts), is targeted into the heavy rafts and thereby can functionally communicate with TCR, albeit less efficiently than when present in the classical lipid rafts [12]. We observed even greater functional difference when comparing the constructs of a critical tyrosine kinase Csk targeted into classical vs. heavy rafts [13].

In the present study, we attempted to identify the part of the LAX molecule responsible for targeting the LAX-LAT constructs into the heavy rafts. To this end, we prepared a series of mutants containing various relevant segments of the LAX sequence and examined their localization in heavy rafts, using the Brij98 DRMs as a proxy for these microdomains. In this respect, the nature of the transmembrane segment appears to be the most important factor.

## Results

In our previous study, we discovered and characterized a novel type of membrane microdomains, denoted here as heavy rafts [12]. However, it remained largely unknown, which features, are responsible for targeting transmembrane proteins to these structures. As shown in our previous paper, the replacement of the extracellular and transmembrane sequences of LAX by those derived from CD25 resulted in targeting into non-raft membrane (solubilized by Brij98). Similar effect was also observed when membrane-proximal basic nonapeptide of LAX (WNWNKRKKR) was replaced by CD3ε-derived sequence (KNRKAKAK). The components of the Brij98 DRMs can be conveniently separated from those present in Brij98-soluble non-raft membrane by the gel filtration on Sepharose 4B [12]. Here, we took advantage of this simple method to identify the part of LAX protein responsible for targeting into heavy rafts.

**Figure 1:**
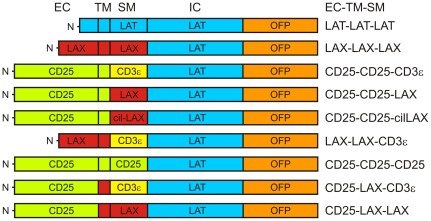
Schematic representation of the chimeric proteins used in this study. EC – extracellular part, TM – transmembrane part, SM – membrane-proximal part, IC – intracellular part, OFP – orange fluorescent protein, N – N-terminus of the protein.

To this end, we prepared several constructs carrying only short sequences originating from LAX. For the sake of consistency with our previous study [12], we derived the new constructs from the previously used constructs CD25-CD25-CD3ε (i.e. containing the extracellular segment of CD25, transmembrane segment of CD25 and cytoplasmic membrane-proximal segment of CD3ε and LAX-LAX-LAX (i.e. containing the extracellular, transmembrane and membrane-proximal fragments of LAX). We also equipped all constructs with the major part of the cytoplasmic domain of LAT followed by Myc-tag and orange fluorescent protein (OFP) (Figure 1). These constructs were used for production of retroviral particles and infection of LAT-deficient J.CaM2.5 cells. The OFP allowed us to sort cells with desired expression levels and to verify the membrane localization by confocal microscopy (not shown).

**Figure 2:**
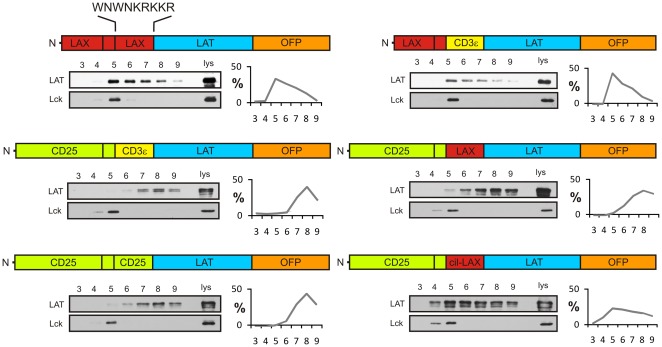
The effect of membrane-proximal sequence of LAX on protein targeting to heavy rafts. Membranes isolated from cells expressing the indicated constructs were solubilized in Brij98, subjected to gel filtration on Sepharose 4B, and analyzed by immunoblotting. The first collected fraction was No. 3, the last one was No. 9. The blots were probed with antibodies to LAT and Lck, a standard marker of lipid rafts. One representative blot is shown for each construct. The results of densitometric analysis were plotted as relative densities of LAT in each fraction. The amino acid sequence of LAX sub-membrane part is provided.

Using this experimental system, we first examined whether the membrane-proximal basic sequence of LAX could be responsible for the targeting of chimeric proteins into heavy rafts. As shown in Figure 2 and consistent with our previous results, the LAX-LAX-LAX construct was highly enriched in the gel filtration fraction No. 5 corresponding to DRMs presumably derived from heavy rafts. Swapping the LAX membrane-proximal peptide for the corresponding sequence of CD3ε (construct LAX-LAX-CD3ε) did not cause any loss of targeting into heavy rafts. The constructs containing extracellular and transmembrane parts of CD25, on the other hand, were present mainly in fractions No. 7, 8, and 9, corresponding to fully solubilized detergent-sensitive non-raft membrane, regardless of the membrane-proximal region used. This also included the CD25-based construct with the membrane-proximal sequence coming from LAX (construct CD25-CD25-LAX). Thus, we concluded that membrane-proximal basic peptide of LAX is not critically involved in localization of LAX-LAT to heavy rafts. Interestingly, the increased detergent resistance of the chimeric CD25 construct containing the membrane-proximal LAX peptide extended by three amino acid residues (CIL) coming from the LAX transmembrane segment (CD25-CD25-cilLAX) suggested that the transmembrane part of LAX could play a more important role.

**Figure 3:**
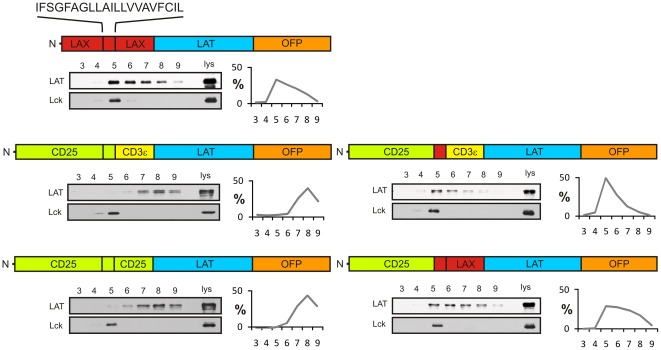
The effect of the transmembrane segment of LAX on targeting to heavy rafts. Membranes isolated from cells expressing the indicated constructs were solubilized in Brij98, subjected to gel filtration on Sepharose 4B, and analyzed by immunoblotting as described in legend to Fig. 2.

Therefore, next we tested this prediction using constructs possessing extracellular part of CD25 and transmembrane part of LAX. As shown in Figure 3, the replacement of CD25 transmembrane domain with the corresponding sequence from LAX (CD25-LAX-CD3ε) caused a major change in the Bri98 solubility of this construct, i.e. its shift from a non-raft to heavy raft membrane. Similarly, a different construct containing extracellular segments of CD25 followed by the LAX transmembrane and membrane-proximal peptides (CD25-LAX-LAX) restored the resistance to Brij98 and localization to heavy rafts.

Collectively, these results provided the evidence that the transmembrane segment of LAX alone is sufficient for targeting of the chimeric constructs to heavy DRMs.

**Figure 4:**
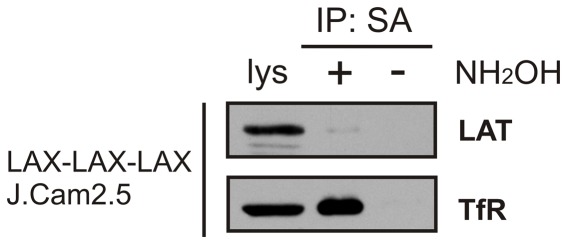
Palmitoylation of LAX-LAX-LAX protein. Protein acylation of the LAX-LAX-LAX protein construct was examined using the acyl-biotinyl exchange chemistry-based method as described in Experimental procedures. Lysate that was not treated with NH_2_OH (hydroxylamine) served as a negative control. A palmitoylated heavy raft protein, transferrin receptor (TfR, CD71), was used as a positive control. IP: SA, material isolated on streptavidin beads.

**Figure 5:**
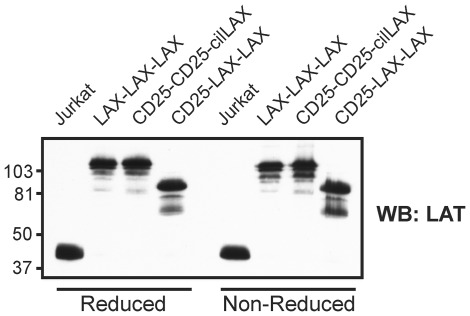
Lack of covalent dimerization of the chimeric protein constructs. Transfectant cell lines expressing the indicated constructs were analyzed by SDS-PAGE under reducing or non-reducing conditions, followed by immunoblotting. Jurkat cells were used as a control expressing endogenous LAT. Positions of m.w. standards (in kDa) are indicated.

LAX contains in its transmembrane domain a cysteine residue that might be, according to the online prediction tool CSS-Palm2.0 [14], potentially acylated (palmitoylated) and this modification might contribute to the LAX targeting to detergent-resistant membrane microdomains. We therefore examined the LAX palmitoylation status by the acyl-biotinyl exchange chemistry-based method that we used successfully before [15]. As shown in Figure 4, we were not able to detect any palmitoylation of the LAX-LAX-LAX construct in the samples prepared from transfected J.CaM2.5 cells. In contrast, we readily detected palmitoylation of another heavy raft protein, transferrin receptor (TfR, CD71), in the same samples. These data indicated that palmitoylation is apparently not involved in LAX targeting to heavy rafts. However, the free thiol group in the distal part of the LAX transmembrane domain could mediate covalent homo- or heterodimerization that might contribute to targeting to heavy rafts. To examine this possibility, we performed SDS-PAGE/western blotting of three relevant transfectants using reduced or non-reduced samples (prepared from cell lysates obtained in the presence of iodoacetamide to prevent any artificial disulfide formation following the cell solubilization). As shown in Figure 5, the mobilities of all three constructs were the same under both reducing and non-reducing conditions, corresponding to monomers. Thus, covalent (disulfide-based) dimerization is obviously not involved in targeting of the constructs to heavy rafts.

## Discussion

In our previous studies [12], [13] we provided biochemical and biological evidence for the existence of specific membrane microdomains, “heavy rafts”, which can be isolated as “heavy DRMs” upon membrane solubilization in Brij98. The heavy rafts are membrane compartments that resist solubilization in Brij98; in contrast to lipid rafts, caveolae or TEMs, heavy DRMs, presumably due to a higher protein to lipid ratio, do not partition into low-density fractions during density gradient ultracentrifugation. The size of the heavy DRMs, as determined by gel filtration on Sepharose 4B, is apparently similar to that of the DRMs derived from classical lipid rafts or TEMs. However, it should be noted that all these DRMs are present in the void volume fractions of Sepharose 4B columns, being therefore too large for size-based separation by this method. Thus, various types of the Brij98-resistant DRMs may significantly differ in their size; such size differences should be revealed e.g. by gel filtration on gels even more porous than Sepharose 4B.

In this study, we addressed the molecular determinants, which are responsible for Brij98 insolubility of a model protein LAX and its targeting to the newly described membrane microdomains, heavy rafts. Protein LAX is a typical transmembrane adaptor protein containing relatively short extracellular part (aa 1–38) and a single transmembrane segment (aa 39–62) followed by a long intracellular sequence (aa 63–398). Interestingly, the cytoplasmic membrane-proximal sequence contains a polybasic motif (KRKKR). A similar cluster of membrane-proximal positively charged amino acid residues in CD4 (RHRRR) is the determinant of its lipid raft localization [16]. To investigate the possibility that the basic motif contributes to LAX targeting into membrane microdomains by mediating interactions with some specific phospholipids and proteins, we prepared (a) several chimeric protein constructs in which the LAX membrane-proximal part was replaced, or (b) CD25-based constructs with inserted LAX membrane-proximal part. However, the LAX membrane-proximal sequence had apparently no effect on targeting of the constructs into heavy rafts.

It has also been shown before that transmembrane domains, protein palmitoylation, association with cholesterol, and protein dimerization can play key roles in targeting of proteins to “classical” lipid rafts. Dimerization controls partitioning to lipid rafts in the case of the urokinase-type plasminogen activator receptor, a GPI-anchored extracellular protein [17]. Some proteins are targeted to lipid rafts by cholesterol recognition/interaction consensus motif located at the junction of transmembrane and cytoplasmic domains (e.g. protein PRiMA [18]) or larger sterol-sensing domains (e.g. HMG-CoA reductase and SCAP) [19]. Protein acylation is necessary for lipid raft targeting of several cytosolic and transmembrane proteins, such as Src kinases [20], G proteins [21], CD46 [22], and CD8β [23]. LAX does not contain cholesterol-binding motifs. In addition, we were not able to detect any palmitoylation of LAX, despite the presence of a cysteine residue fulfilling the criteria for a potential palmitoylation site. As shown in Figure 5, no covalent dimerization of our constructs containing the unmodified cysteine was detectable that might contribute to targeting to heavy rafts. However, non-covalent oligomerization of LAX or LAX-derived constructs cannot be excluded by our present results.

Intact transmembrane segments are required for the lipid raft localization of proteins like CD44 [24] or CD40 [25]. Thus, we investigated the role of LAX transmembrane part in targeting to heavy rafts. We constructed several proteins differing from each other just by the presence or absence of the transmembrane part of LAX. We isolated membranes, solubilized them in Bri98-containing buffer and subjected them to gel filtration. Using this approach, we could show that the transmembrane segment of LAX is the main determinant of the heavy raft targeting. The properties of the construct CD25-CD25-cilLAX also indicated that the inner leaflet-proximal part of the LAX transmembrane segment, namely the short sequence CIL, is apparently critical in this respect. At this point we are not able to define in more detail the necessary and sufficient features of the LAX transmembrane domain responsible for the observed heavy raft targeting. Also, it remains to be examined whether other transmembrane proteins present in heavy rafts (e.g. CD28, CD5, CD45, MHCI [12]) are also targeted to these microdomains solely via their TM domains or if some of them (e.g. TfR) are dependent on lipid modifications or cholesterol binding.

In spite of the currently frequent opinion that the use of detergents is not adequate for membrane microdomain research, we believe that this approach is a very useful and relevant tool for studying various functional aspects of membrane proteins and microdomains. We believe that Brij98, in contrast to the mostly used Triton X-100, may be an optimal detergent in this respect, as it produces stable products relatively independent of the conditions used (concentration of the detergent, time of solubilization, temperature), which may be close to the ideal situation shown by Lichtenberg et al. (Figure 1 in [26]). Our results refute possible objections that Brij98 simply does not solubilize plasma membrane proteins effectively. In fact, the replacement of LAX transmembrane segment with the one coming from CD25 clearly resulted in sensitivity to solubilization by Brij98. On the other hand, replacement of the transmembrane part of CD25 by the LAX transmembrane segment markedly shifted the protein to fractions containing large DRMs. This indicates that the nature of the protein rather than detergent properties determine to which types of DRMs are the proteins partitioned.

To conclude, we identified the transmembrane part of LAX to be responsible for targeting of LAX constructs to Brij98-resistant heavy raft microdomains. Further research should be directed to identification of molecular features responsible for targeting of other proteins to heavy rafts and to elucidation of functional roles of these microdomains.

## Materials and Methods

### DNA constructs and DNA cloning

All constructs used in this study are schematically depicted in Figure 1. For the maximal simplicity, the constructs are logically named according to the extracellular, transmembrane, and membrane-proximal parts (EC-TM-SM) they are composed of. Transmembrane segment boundaries were determined using the online prediction tool, TMHMM Server v.2.0 [27]. The constructs LAT-LAT-LAT, LAX-LAX-LAX, and CD25-CD25-CD3ε were already used previously [12]. Other constructs were generated by PCR and ligation of enzyme restriction fragments and verified by automated sequencing. Extracellular parts comprised human CD25 (aa 1–240) and LAX (aa 1–37), transmembrane parts comprised human CD25 (aa 241–259) and LAX (aa 38–58), and cytoplasmic membrane-proximal parts comprised human CD25 (aa 260–270), LAX (aa 59–67 or aa 56–67, in cilLAX), and CD3ε (aa 153–160). The intracellular part of human LAT (aa 34–233) was present in all constructs. The amino acid residues are numbered according to GenBank protein reference sequences CD25 (NP_000408), LAX (NP_060243), CD3ε (NP_000724), and LAT (NP_001014987). The coding sequences of all chimeric constructs were fused to the coding sequences of Myc-tag and orange fluorescent protein (OFP) in pMXsIN retroviral vector backbone as described previously [12].

### Antibodies

Rabbit antiserum to human LAT [8] was kindly provided by Dr. L. Samelson. Mouse mAb to Lck (LCK-01) and mAb to CD71/transferrin receptor (MEM-189) were obtained from Exbio (Vestec, Czech Republic).

### Cells and cell culture

The human LAT-negative J.CaM2.5 lymphoma cells [28] were kindly provided by Dr. A.Weiss. These cells were cultured in RPMI 1640 supplemented with 10% FCS (Biochrom AG, Berlin, Germany) and antibiotics at 37°C in 5% CO_2_. Phoenix-Ampho cells (Orbigen, San Diego, CA) used for the production of retroviruses were cultured in DMEM supplemented with 10% FCS and antibiotics at 37°C in 5% CO_2_.

### Retroviral infection and MACS sorting

Retroviruses were produced in Phoenix-Ampho cells transfected with plasmid DNA using Lipofectamine 2000 (Invitrogen). After 48 h, retroviral supernatant was collected and centrifuged to remove debris and then used to spin-infect (1,200× g/90 min at RT) J.CaM2.5 cells in the presence of Polybrene (4 µg/ml, Sigma). Cells were allowed to expand in culture for one week and the infected (OFP expressing) cells were enriched by FACS sorting.

### Plasma membrane preparation

Cells (2×10^7^) were resuspended in 1 ml of ice-cold hypotonic buffer (10 mM HEPES pH 7.4, 42 mM KCl, 5 mM MgCl_2_, mixture of protease inhibitors [Complete, Roche]), incubated on ice for 15 min and then passed 10× through the 25-gauge needle. The suspension was centrifuged 5 min at 200× g and 4°C to remove nuclei. The ice-cold post-nuclear supernatant was centrifuged 10 min. at 25,000× g and 2°C to pellet the membranes.

### Preparation of membrane lysates

Cell membranes were lysed in 300 µl of lysis buffer (20 mM Tris pH 8.0, 100 mM NaCl, 5 mM iodoacetamide, 10 mM EDTA, 50 mM NaF, 10 mM Na_4_P_2_O_7_, 10% v/v glycerol and protease inhibitors) containing 1% w/v detergent Brij98 (Sigma), 30 min on ice. To remove insoluble debris, the lysate was spun down at 16,000× g for 5 min at 4°C.

### Gel filtration

All steps were performed at 4°C. The membrane lysate (100 µl) was applied at the top of a 1 ml Sepharose 4B column (equilibrated with the lysis buffer with the detergent) and washed with the lysis buffer; 100 µl fractions were collected and analyzed by SDS-PAGE and western blotting performed essentially as described before [29], Typical lipid raft proteins (e.g. Lck) were eluted in fractions 4 and 5 containing very large complexes. Immunoblots were quantified using densitometry software (Aida Image Analyzer). Statistical evaluations were done and plots were created in Excel (Microsoft).

### Protein acylation analysis

Acylation (palmitoylation) of LAX was examined using the acyl-biotinyl exchange chemistry-based method [30]. Briefly, plasma membranes from 5×10^7^ cells were isolated, and acyl (palmitate) protein modifications were removed by hydroxylamine and replaced with biotin. Biotinylated proteins were then isolated on streptavidin agarose beads and analyzed by immunoblotting.

### Examination of potential covalent dimerization of the chimeric proteins

Transfectant cell lines expressing the informative constructs (all containing the critical LAX cysteine residue) were detergent-solubilized in lysis buffer containing 1% detergent dodecylmaltoside and 5 mM iodoacetamide, the lysates were mixed 1∶1 with reducing or non-reducing 2× sample buffer, boiled, SDS-PAGE on 10% gel was performed followed by western blotting; the constructs were detected by anti-LAT antibody.
